# Technical Note: Proof of concept for radiomics‐based quality assurance for computed tomography

**DOI:** 10.1002/acm2.12750

**Published:** 2019-10-14

**Authors:** Luciano R. F. Branco, Rachel B. Ger, Dennis S. Mackin, Shouhao Zhou, Laurence E. Court, Rick R. Layman

**Affiliations:** ^1^ Department of Radiation Physics The University of Texas MD Anderson Cancer Center Houston TX USA; ^2^ MD Anderson Cancer Center UTHealth Graduate School of Biomedical Sciences Houston TX USA; ^3^ Department of Biostatistics The University of Texas MD Anderson Cancer Center Houston TX USA; ^4^ Departments of Imaging Physics The University of Texas MD Anderson Cancer Center Houston TX USA

**Keywords:** CT, QA, quantitative imaging, radiomics

## Abstract

**Purpose:**

Routine quality assurance (QA) testing to identify malfunctions in medical imaging devices is a standard practice and plays an important role in meeting quality standards. However, current daily computed tomography (CT) QA techniques have proven to be inadequate for the detection of subtle artifacts on scans. Therefore, we investigated the ability of a radiomics phantom to detect subtle artifacts not detected in conventional daily QA.

**Methods:**

An updated credence cartridge radiomics phantom was used in this study, with a focus on two of the cartridges (rubber and cork) in the phantom. The phantom was scanned using a Siemens Definition Flash CT scanner, which was reported to produce a subtle line pattern artifact. Images were then imported into the IBEX software program, and 49 features were extracted from the two cartridges using four different preprocessing techniques. Each feature was then compared with features for the same scanner several months previously and with features from controlled CT scans obtained using 100 scanners.

**Results:**

Of 196 total features for the test scanner, 79 (40%) from the rubber cartridge and 70 (36%) from the cork cartridge were three or more standard deviations away from the mean of the controlled scan population data. Feature values for the artifact‐producing scanner were closer to the population mean when features were preprocessed with Butterworth smoothing. The feature most sensitive to the artifact was co‐occurrence matrix maximum probability. The deviation from the mean for this feature was more than seven times greater when the scanner was malfunctioning (7.56 versus 1.01).

**Conclusions:**

Radiomics features extracted from a texture phantom were able to identify an artifact‐producing scanner as an outlier among 100 CT scanners. This preliminary analysis demonstrated the potential of radiomics in CT QA to identify subtle artifacts not detected using the currently employed daily QA techniques.

## 1 INTRODUCTION

Quality assurance (QA) testing is a widely used method of detecting malfunctions in medical imaging devices such as computed tomography (CT) scanners. Therefore, the QA process plays an important role in meeting quality standards and ensuring good image quality. QA is traditionally carried out following state‐specific requirements or recommendations from accrediting bodies (e.g. American College of Radiology) or the scanner manufacturer with a standard phantom for which simple metrics, such as uniformity, are calculated.^1^ However, whereas daily QA has been able to detect calibration issues, it is less effective at identifying subtle artifacts.^2^, ^3^ Although subtle, these artifacts can cause issues for diagnostic scans and potentially indicate a significant underlying issue regarding system performance. In practice, detection of subtle artifacts is often dependent on the experience of radiologists evaluating the images and missed detections may cause the patient to have additional unplanned scans, resulting in excess dose.^4^

Radiomics, in which voxel relationships are evaluated to identify textural patterns, has shown promise in separating patients into low‐ and high‐risk groups for assessment of survival.^5^, ^6^, ^7^, ^8^, ^9^ This separation of patients demonstrates the ability of radiomics features to identify small textural differences on CT images. We hypothesized that applying this radiomics analysis to CT QA could allow for determination of small textural differences on images with subtle artifacts not detected in conventional QA. We tested this hypothesis by performing a controlled CT scan of a radiomics phantom using a scanner producing a subtle artifact not detected in conventional QA testing.

## 2 MATERIALS AND METHODS

An updated credence cartridge radiomics phantom was used as we described previously^10^ to acquire the CT scan. This phantom consists of six cartridges composed of different materials that produce a spectrum of textures. For this study, two of the cartridges—one composed of rubber and one composed of cork—were investigated because they have been shown to produce textures most similar to those on images of nonsmall cell lung tumors.^11^

**Figure 1:**
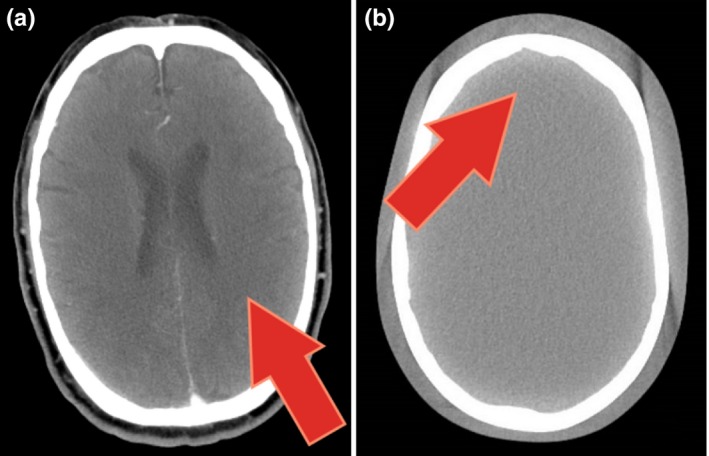
CT scans with subtle artifacts not detected during daily QA. The red arrows point to subtle line pattern artifacts. (a) Patient lymphoma scan. (b) An anthropomorphic phantom scan performed after the artifact was detected.

For this study, the phantom was imaged using a Siemens SOMATOM Definition Flash CT scanner (Siemens Healthineers, Forchheim, Germany). This scanner produced an artifact with a line pattern that was identified by a radiologist reviewing a patient’s CT lymphoma study [Fig. 1(a)]. In addition, after the artifact was detected, the same scanner was used to image an anthropomorphic phantom, demonstrating the same line pattern [Fig. 1(b)]. This particular artifact was not observed in other types of scans.

Routine daily QA is performed as per manufacturer specifications on the system since it was installed in July 2013. The manufacturer provided phantom is scanned using the follow acquisition technique: 120 kVp, 230 mAs, pitch 0.6, 25 cm display field of view, image and interval thickness of 5 × 5 mm, and B30f convolution kernel. A 2 cm diameter circular region of interest (ROI) is placed centrally in the middle image for the water insert of the phantom. The Hounsfield unit (HU) of water and standard deviation are recorded as a surrogate for image noise. Four additional ROIs of the same size (2 cm diameter) are placed peripherally at 12, 3, 6, and 9 o’clock position to measure uniformity. The difference between the mean value of each peripheral and central ROI is calculated and recorded. In addition, the uniformity images are visually reviewed for artifacts by either the Qualified Medical Physicist or trained support staff.

**Figure 2:**
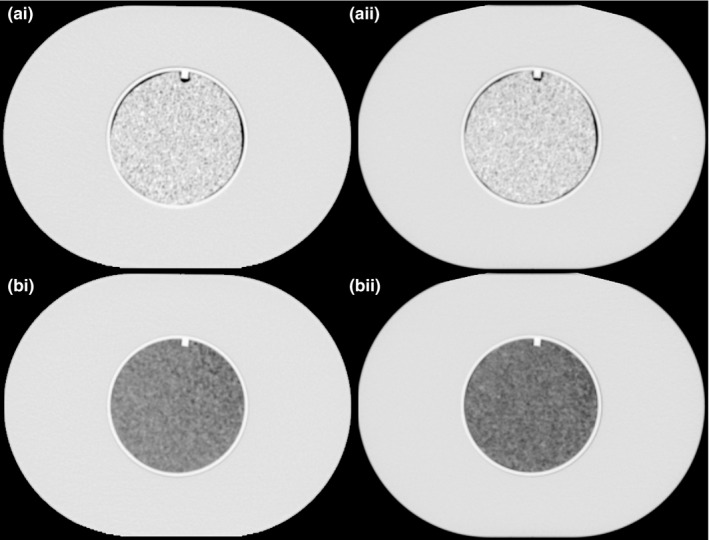
CT scans of the radiomics phantom used in this study. To the left (i‐a and i‐b) are the scans referred to first time point and to the right (ii‐a and ii‐b) is the second time point in this study. Top two scans (i‐a and ii‐a) are the cartridges containing rubber and the two bottom ones (i‐b and ii‐b) have cartridges with the material cork.

To facilitate direct comparison of the Flash CT scanner with other CT scanners the same scanner protocol and settings employed for a population of controlled scans that we described previously^10^ were used. This population consisted of controlled scans taken using 100 CT scanners. The controlled protocol was specific for each vendor and designed to minimize feature differences across vendors. Therefore, this eliminates scanning protocol variabilities, as there is only one protocol. For this dual source Flash CT scanner, only tube A was used with the following acquisition parameters: 120 kVp, 200 mAs, pitch 1, 50 cm display field of view, image and interval thickness of 3 × 3 mm, and B31s convolution kernel. Additionally, the data set also contained images obtained using the same scanner investigated in this study but at an earlier time point when no artifacts were reported. This datum was referred to as our first time point in this study, whereas the new scan – in which the artifact was reported – was called the second time point. Images from the phantom in the first and second time points are presented in Fig. 2.

All images were imported into IBEX, a freely available radiomics software program.^12^, ^13^ ROIs with an 8.2 cm diameter and 1.9 cm height were semiautomatically contoured for the rubber and cork cartridges using an in‐house MATLAB script (MathWorks, Natick, MA). A total of 49 features were extracted from images of the two cartridges. All of the features examined were listed previously^10^ and consisted of 22 gray‐level co‐occurrence matrix (GLCM), 11 gray‐level run length matrix (GLRLM), five neighborhood gray tone difference matrix (NGTDM), and 11 intensity histogram features. The features were calculated using four preprocessing techniques: 1) no preprocessing, 2) 8‐bit depth rescaling, 3) Butterworth smoothing, and 4) Butterworth smoothing and 8‐bit depth rescaling. These preprocessing techniques were chosen because in studies of nonsmall cell lung cancer survival, different features were shown to be the most prognostic when using different preprocessing techniques.^8^, ^14^ The settings for the features and preprocessing are detailed in the supplemental material reported by Fave et al.^8^

To determine whether a given feature calculated on the second time point scan can identify the scan as an outlier (i.e., a surrogate for detecting an artifact in the scan in this study), the mean and standard deviation of the population of controlled scans for each combination of feature and preprocessing algorithm were calculated. For each feature value, the number of standard deviations of a given time point scan away from the mean of the controlled scan population (*N*_sd_ *_,i_*) was calculated using the formula.

$$N_{\text{sd},i}=\frac{\bar{f_{p}}-f_{i}}{\sigma_{p}},$$

in which* i* is the time point (1 for the first time point and 2 for the second time point), $\bar{f_{p}}$ is the average feature value for the controlled scan population, $f_{i}$ is the feature value from the scan at *i*, and $\sigma_{p}$ is the standard deviation of the feature value in the scan population. Features from the second time point with the largest standard deviations from the population mean and therefore the leading ones for identifying the second time point as an outlier were selected for further investigation. The Wilcoxon signed rank test was performed to determine whether *N*_sd,2_ for each feature was the same when extracted from the cork and rubber cartridges. The Wilcoxon signed rank test was also performed to compare *N*_sd,2_ values across different preprocessing techniques. This statistical test was used for these comparisons because the normality of the data could not be established using the Shapiro‐Wilk test. *P* values below 0.05 were considered statistically significant. All analyses were conducted with the R computing language (version 3.4.1).

## 3 RESULTS

**Figure 3:**
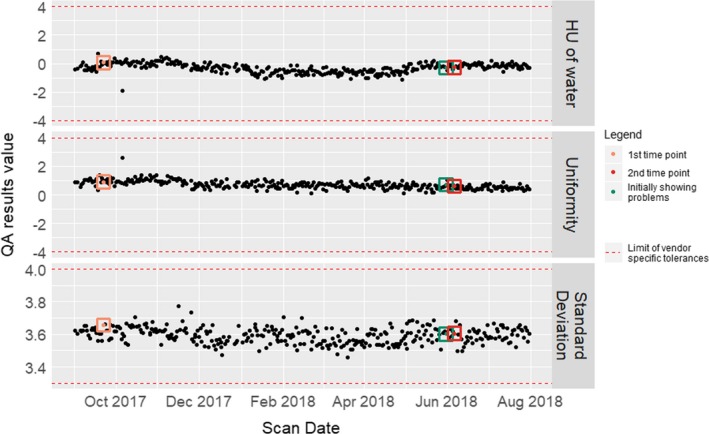
Daily QA parameters extracted from the Siemens SOMOTOM Definition Flash CT Scanner. Three parameters were extracted in daily QA testing performed from 1 September 2017 to 31 July 2018: HU of water, uniformity, and standard deviation. The highlighted time points shown in orange, red, and green represent 1) the designated first time point in this study (i.e., our reference scan for comparison), 2) the designated second time point in this study (i.e., the radiomics phantom scan performed when the scanner was producing an artifact), and 3) when the artifact was first identified by the radiologist, respectively. The horizontal red lines show the limit values in the scanner meeting vendor‐specific tolerances and therefore passing the daily QA testing.

The artifact shown in Fig. 1 was not detected by the daily QA performed as per manufacturer’s specifications, as seen in Fig. 3, in which no trend in daily QA metrics can be clearly discerned and therefore associated with any problems with the scanner. All daily QA values, HU of water, uniformity, and standard deviation, fell within the tolerance limits. Additionally, the visual review of uniformity detected no artifacts.

**Figure 4:**
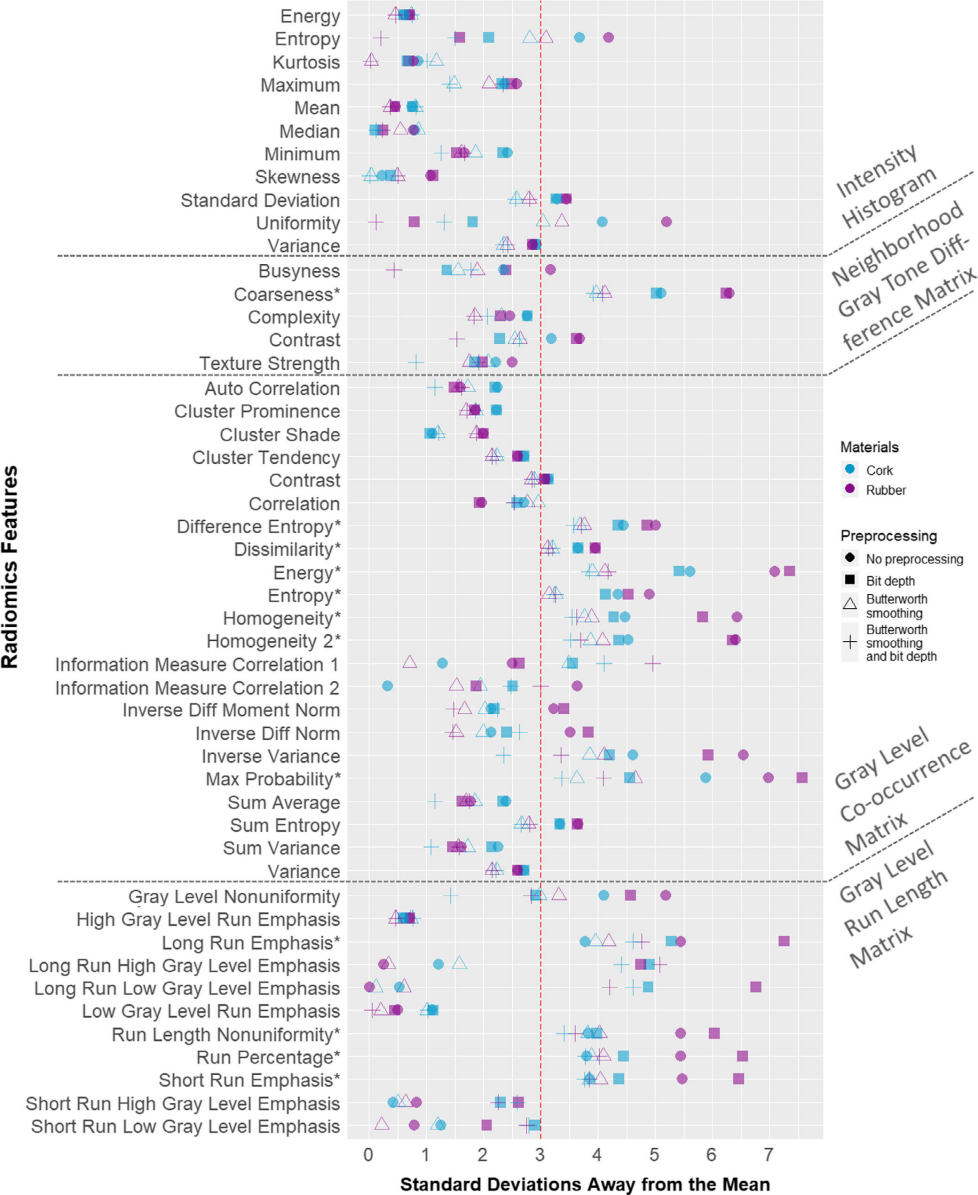
Standard deviations away from the mean of 100 CT scans in the controlled population for all features. The distance from the mean of the population of controlled scans is shown for the second time point scan, which produced an artifact not detected in daily QA testing. Each feature value is presented as measured in both cartridges: blue for cork and purple for rubber. Additionally, the four preprocessing techniques are represented by different symbols: circle, no preprocessing; square, 8‐bit depth rescaling; triangle, Butterworth smoothing; +, Butterworth smoothing and 8‐bit depth rescaling. *Feature that produced an *N*_sd,2_ of at least three across all preprocessing techniques. QA, quality assurance.

For the rubber and cork cartridges**,** of a total of 196 feature‐preprocessing combinations, the *N*_sd,2_ was greater than or equal to three in 79 (40%) and 70 (36%) of cases, respectively. Of all features, 12 had an *N*_sd,2_ of three or greater for all four preprocessing algorithms: seven from GLCM, four from GLRLM, and one from NGTDM. N_sd,2_ values for each feature are shown in Fig. 4.

Fifty‐eight percent of the features had a higher *N*_sd,2_ in rubber than in cork. Also, Wilcoxon signed rank test results demonstrated that the *N*_sd,2_ was larger for features in rubber than for those in cork (*P* = 0.001).

When comparing the different preprocessing techniques, the Wilcoxon signed rank test findings demonstrated that the *N*_sd_ *_,2_* values were larger when no preprocessing was used versus Butterworth smoothing (*P* = 5 × 10^−12^) or versus Butterworth smoothing and bit depth rescaling (*P* = 10^−6^). The *N*_sd_ *_,2_* values were also larger when bit depth rescaling was used versus Butterworth smoothing (*P* = 10^−10^) or versus Butterworth smoothing and bit depth rescaling (*P* = 4 × 10^−11^). However, we did not see a statistical difference in the *N*_sd_ *_,2_* values between no preprocessing and bit depth rescaling (*P* = 0.17) or between Butterworth smoothing and Butterworth smoothing and bit depth rescaling (*P* = 0.11).

**Figure 5:**
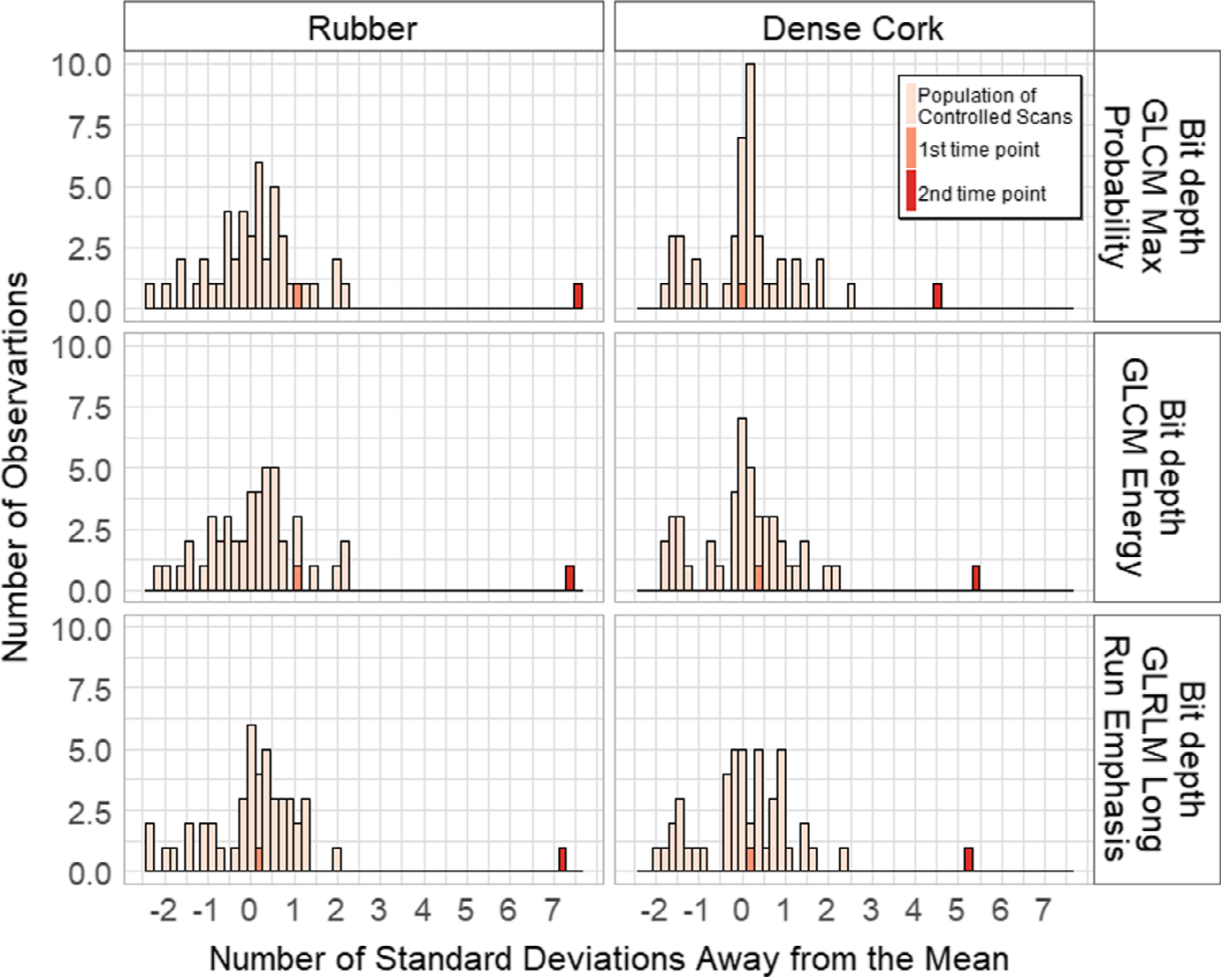
Histograms of feature value distributions in all scans. The top three feature‐preprocessing combinations identifying the second time point as an outlier are shown. The three features all used 8‐bit depth rescaling. The features selected were maximum probability from gray‐level co‐occurrence matrix (GLCM), energy from GLCM, and long run emphasis from gray‐level run length matrix (GLRLM). The first time point is inside the controlled scan population distribution, whereas the second time point is outside.

To examine the relationship of the first and second time point scans with the controlled scan population, we selected three feature‐preprocessing combinations with the largest *N*_sd_ *_,2_* values. The three feature‐preprocessing combinations selected were: maximum probability with bit depth rescaling from GLCM, energy with bit depth rescaling from GLCM, and long run emphasis with bit depth rescaling from GLRLM. Histograms of these feature values in all of the scans are shown in Fig. 5. For the rubber cartridge, the *N*_sd,1_ and *N*_sd,2_ of maximum probability were 1.01 and 7.56, respectively; the *N*_sd,1_ and *N*_sd,2_ of energy were 1.05 and 7.34, respectively; and the *N*_sd,1_ and *N*_sd,2_ of long run emphasis were 0.25 and 7.26, respectively. For the cork cartridge, the *N*_sd,1_ and *N*_sd,2_ of maximum probability were 0.07 and 4.56, respectively; the *N*_sd,1_ and *N*_sd,2_ of energy were 0.31 and 5.41, respectively; and the *N*_sd,1_ and *N*_sd,2_ of long run emphasis were 0.15 and 5.28, respectively.

## 4 DISCUSSION

We investigated the ability of radiomics features to detect a subtle artifact in a CT scan –only resolved once the x‐ray tube was replaced – that was not detected in conventional daily QA testing. We identified several features that varied considerably when measured in a radiomics phantom using a scanner that produced a subtle artifact compared with a population of scanners using a controlled protocol. Additionally, the proposed method was capable of identifying an outlier by using a controlled protocol without the need of using the singular CT imaging technique that was producing the artifact in the clinic. This proves to be useful in a clinical scenario since only the controlled protocol scan is needed for the analysis.

The values for almost half of the features deviated greatly in the images from the second time point when compared with the population of controlled CT scans. Twelve features measured at the second time point were three or more standard deviations away from the controlled scan population mean when measured in both the rubber and cork cartridges across all four preprocessing algorithms. The top three features that had the largest *N*_sd_ *_,2_* came from two different feature categories: GLCM and GLRLM. This demonstrated that the artifact impacted second‐order statistics and therefore affected the spatial relationship of HUs in the phantom. However, this difference did not noticeably affect the uniformity, linearity, or HU of water as measured in daily QA. This lack of detection of the artifact in daily QA is consistent with our results demonstrating that intensity histogram values were least affected by the subtle artifact.

Standard deviation values of the second time point were significantly higher for features extracted with no preprocessing or bit depth rescaling than for features extracted with Butterworth smoothing alone or Butterworth smoothing and bit depth rescaling. This suggests that the Butterworth filter smoothens the textural differences for this particular subtle artifact.

This study had several limitations. We did not perform longitudinal scans, which would have allowed tracking of the features from the scanner from when they were within the population and there was no artifact, to when they were outside the population and there was an artifact. However, as previous studies have shown that radiomics features from CT scans are reproducible over time,^15^, ^16^ we are convinced that this result is due to the artifact and not due to random fluctuations in feature values in repeat measurements. Likewise, while the first time point demonstrated that the features from the scanner images were within the population of scans obtained using the 100 CT scanners and the features from the second time point were outside the population’s distribution, a trend in feature value cannot be established using only these two time points. Additionally, we performed all artifact analyses using only one scanner. However, this was a feasibility study and further investigation using multiple scanners would be required to better elucidate the relationship of detection of artifacts with radiomics features and before any clinical usage.

Another limitation of this study is that we only tested two materials – the ones from the phantom which most closely produce textures similar to textures from patients. The use of more materials could provide us with a more complete picture of the relation between radiomics features and the textural differences of a CT artifact. However, features that are not similar to those from patients may erroneously identify issues, as they do not have similar values to patients.

Even with these limitations, the results of this study are promising, specifically, the ability of features extracted from a controlled CT scan of a radiomics phantom to identify an artifact‐producing scanner as very far away from the controlled scan population distribution of feature values. In a clinical environment, this could lead to better QA than waiting for a radiologist to report the artifacts within a patient’s scan. Additionally, this method would allow for the analysis of the extracted radiomics features relative to the controlled scan population distribution to be done automatically without the requirement of visual inspection of scans to identify artifacts. There is still much left to prove with this method by tracking scanners over time to determine the existence of a trend in feature values as well as determining whether the same features are able to identify multiple scanners producing artifacts as outliers (i.e., scanners producing artifacts). However, this proof‐of‐concept study is promising for extending radiomics to the routine quality assurance of CT scanners.

## 5 CONCLUSION

In this study, we found that features from a radiomics phantom identified an artifact‐producing CT scanner as an outlier relative to a population of 100 scanners after imaging the same phantom using a controlled protocol. This preliminary study demonstrates the potential for radiomics in CT QA to identify subtle artifacts not detected using current daily QA techniques. The radiomics phantom methodology presented herein can contribute to further investigation of radiomics features being extended to QA practices.

## CONFLICT OF INTEREST

The authors declare no conflict of interests.
